# Effect of simultaneous testing of two mice in the tail suspension test and forced swim test

**DOI:** 10.1038/s41598-022-12986-9

**Published:** 2022-06-02

**Authors:** Hiroshi Ueno, Yu Takahashi, Shinji Murakami, Kenta Wani, Yosuke Matsumoto, Motoi Okamoto, Takeshi Ishihara

**Affiliations:** 1 Department of Medical Technology, Kawasaki University of Medical Welfare, 288, Matsushima, Kurashiki, Okayama, 701-0193 Japan; 2 Department of Psychiatry, Kawasaki Medical School, Kurashiki, 701-0192 Japan; 3 Department of Neuropsychiatry, Graduate School of Medicine, Dentistry and Pharmaceutical Sciences, Okayama University, Okayama, 700-8558 Japan; 4 Department of Medical Technology, Graduate School of Health Sciences, Okayama University, Okayama, 700-8558 Japan

**Keywords:** Neuroscience, Neurology

## Abstract

In mouse studies, the results of behavioural experiments are greatly affected by differences in the experimental environment and handling methods. The Porsolt forced swim test and tail suspension test are widely used to evaluate predictive models of depression-like behaviour in mice. It has not been clarified how the results of these tests are affected by testing single or multiple mice simultaneously. Therefore, this study evaluated the differences between testing two mice simultaneously or separately. To investigate the effect of testing multiple mice simultaneously, the Porsolt forced swim test and tail suspension test were performed in three patterns: (1) testing with an opaque partition between two mice, (2) testing without a partition between two mice, and (3) testing a single mouse. In the Porsolt forced swim test, the mice tested simultaneously without a partition demonstrated increased immobility time as compared to mice tested alone. No difference in immobility time was observed between the three groups in the tail suspension test. Our results showed that the environment of behavioural experiments investigating depression-like behaviour in mice can cause a difference in depression-like behaviour. The results of this experiment indicated that it is necessary to describe the method used for behavioural testing in detail.

Mice have been the most widely used animals for experiments on disease, behaviour, and pharmacology studies over the past century^1^. To transfer the results obtained from mouse experiments to humans successfully, it is necessary to determine the appropriate treatment, and handling and experimental methods to be used in mice.

In general, depression-like behaviour, aggression, activity, anxiety-like behaviour, and other types of behaviour, are measured in mice through a series of behavioural tests in order to analyse the effects of drugs and other stimuli^2^. Behavioural test batteries have been used in cohorts of inbred and mutant mouse strains to assess a variety of behavioural characteristics, such as motor activity, sensory and motor function, anxiety-like behaviour, learning, and memory^3,4^. Therefore, behavioural tests are generally designed to minimise the potential effects of various confounding factors. The use of a set of standardised behavioural tests is required to ensure a more accurate interpretation of behavioural phenotypes.

The tail suspension and forced swim tests are among the tests used in behavioural test batteries. These tests have been used for decades to assess depressive-like behaviour in mice and rats^5^. The tail suspension test is a method in which a mouse or rat is hung by attaching its tail to a box or rod using adhesive tape or similar material^6^. The behaviour obtained is recorded, and depressive-like behaviour is estimated by the time the suspended animal remains immobile. The equipment used in the forced swim test consists of a transparent cylinder. The cylinder is filled with water to the extent that the legs of the mouse or rat cannot touch the bottom surface of the cylinder, and the mouse or rat is placed in the water to record the behaviour^7^. Depression-like behaviour is again estimated by the total time during which the animal remains immobile.

It is generally accepted that the tail suspension test and the forced swim test are well established. However, some issues remain to be addressed. For instance, it is not clear how the results for individual experimental animals are affected by conducting the tests at different times and places, or how they are influenced by testing several experimental animals simultaneously. Currently, some commercially available behavioural test devices can be used with multiple mice simultaneously, while others can only be used to test a single mouse at a time. It is therefore important to determine the effect that this difference in the experimental method and in the experimental environment has on the results of these tests.

Mice use visual and olfactory information to show interest in the abnormal behaviour of conspecifics^8–10^. Mice visually recognise and show interest in cage mates that demonstrate abnormal behaviours^11^. Furthermore, it has been reported that mice can discriminate between photographs^12^. In addition, recent studies have shown that they can recognise virtual reality spaces^13,14^. Other studies have shown that mice are social animals with a strong tendency to follow allogeneic individuals^15^. These reports show that mice change their behaviour based on visual, olfactory, and auditory information, suggesting that there is a high possibility that the experimental animals’ behaviour could change in response to the presence of other individuals when multiple individuals simultaneously undergo the tail suspension test or the forced swim test. In fact, some reports have recommended taking steps to prevent individual animals from observing conspecifics being tested, although the rationale for this was not explained^6^.

In this study we tested C57BL/6 mice, an inbred strain widely used for knockout and transgenic models^16^. Behavioural phenotypes in genetically engineered mice are characterized through a series of behavioural experiments.

The purpose of this study was to determine the proper environment for performing the forced swim and tail suspension tests in the analysis of depressive-like behaviour. We sought to determine potential differences between testing two individual mice simultaneously or independently from each other.

## Materials and methods

### Animals

All animal experiments were performed in accordance with the ARRIVE guidelines (http://www.nc3rs.org.uk/arrive-guidelines) and the U.S. National Institutes of Health (NIH) Guide for the Care and Use of Laboratory Animals (NIH Publication No. 80–23, revised in 1996) and were approved by the Committee for Animal Experiments at Kawasaki Medical School Advanced Research Centre. All efforts were made to minimise the number of animals used and their suffering. The required sample size was calculated by a power analysis before the start of the experiment.

C57BL/6 N male mice (age: 10 weeks) were purchased from Charles River Laboratories (Kanagawa, Japan) and were housed in cages (four to five animals per cage) with food and water provided ad libitum under a 12 h light/dark cycle at 23–26 °C. Considering that behavioural diversity is partially sex-dependent, and comparing the behaviour of males versus females was not the purpose of this experiment, only male mice were included in the study.

### Behavioural tests

All behavioural tests were conducted in behavioural testing rooms, between 09:00 and 16:00 during the light phase of the light/dark cycle. After the tests, the equipment and toys were cleaned with 70% ethanol and super-hypochlorous water to prevent artefacts caused by lingering olfactory cues. Behavioural tests were performed in naïve mice in accordance with the test order described below. Mice were randomly divided (http://www.randomizer.org) into two groups: a demonstrator and an observer (test mice). Cage mates were used as demonstrator mice. At the end of the study, the animals were sacrificed by CO_2_ inhalation.

### Tail suspension test

**Figure 1:**
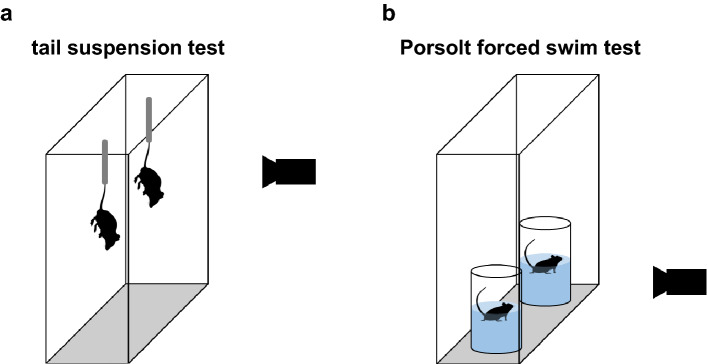
Schematic illustration of the experimental apparatus. (**a)** Tail suspension test. (**b**) Porsolt forced swim test.

Depression-like behaviour was examined using the tail suspension test, as previously described^17–19^. The test apparatus consisted of white acrylic walls (20 × 40 × 60 cm), with one open wall through which the animals could be video recorded. The centre of the apparatus was illuminated with 250 lx. Two animals could be tested at the same time (Fig. 1a), and they could be separated using an opaque partition placed in the centre of the apparatus. Each mouse was suspended by the tail 60 cm above the floor of the chamber using adhesive tape placed < 1 cm from the tip of the tail. The resultant behaviour was recorded by a video camera for 6 min. The behaviour was later analysed to determine the following parameters: number of immobility episodes and total duration of immobility. The total amount of time during which each mouse remained immobile was recorded in seconds and then expressed as a percentage of total time or as a percentage per minute. In this test, the ‘immobile period’ was defined as the period when the animals stopped struggling for ≥ 1 s. Data acquisition and analysis were performed using a video tracking software (ANY-MAZE, Stoelting Co., Wood Dale, IL, USA).

**Figure 2:**
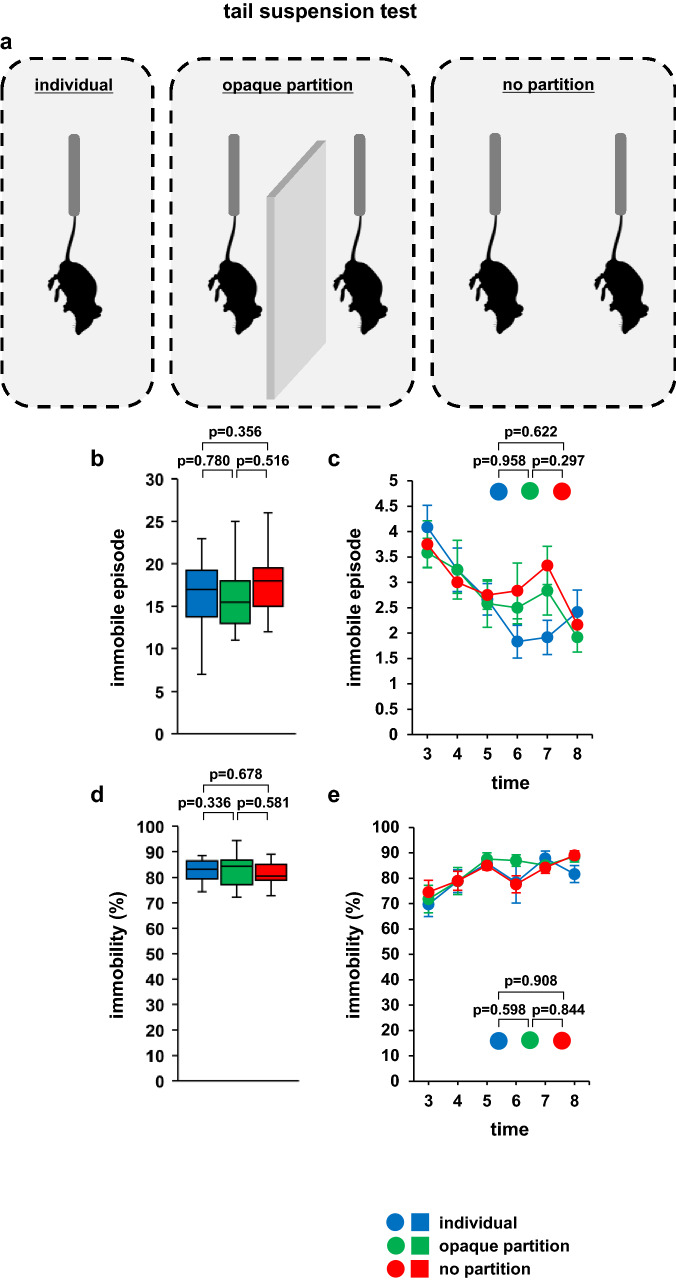
Simultaneous tail suspension testing of two mice. (**a**) Schematic diagram of the experiment. Graphs showing the total number of immobility episodes (**b**) and the immobility episodes during each 1-min period (**c**). Graphs showing the total time spent immobile (**d**) and the proportion of time spent immobile during each 1-min period (**e**). All data are presented as box plots (**b**, **d**) or means ± standard errors (**c**, **e**). Statistical significance is represented by asterisks: *p < 0.05, ^+^*p* < 0.1. The *p* values were calculated using one-way analysis of variance (analysis of variance [ANOVA] in (**b**) and (**d**) and two-way repeated-measures ANOVA in (**c**) and (**e**). SEM: standard error of the mean. individual: n = 10, opaque partition: n = 10, no partition: n = 10. Respective mean and SEM are listed in Table 1.

Three patterns were used to assess whether the behaviour of the test subjects changed depending on the experimental conditions: (1) only a single mouse tested at a time (individual group), (2) presence of an opaque partition plate separating two cage mates being tested simultaneously (opaque partition group), and (3) two cage mates tested simultaneously, without the partition plate (no-partition group) (Fig. 2a).

### Porsolt forced swim test

The Porsolt forced swim test was also used to examine depression-like behaviour. The apparatus consisted of four Plexiglas cylinders (20 cm [height] × 10 cm [diameter]). Each cylinder was placed in the centre of the apparatus, consisting of a square area surrounded by white acrylic walls (20 × 40 × 60 cm), with one wall open through which the behaviour was recorded (Fig. 1b). The centre of the apparatus was illuminated with 250 lx. This apparatus could also be used with an opaque partition at the centre. Only one wall of the square area surrounded by the white acrylic wall was open and we took a video of the mouse from here. The cylinders were filled with water (23 °C) to a depth of 7.5 cm, based on previous studies^20,21^. The mice were placed in the cylinders for 6 min and video recorded. The behaviour of the mice was analysed to determine the following parameters: the number of immobility episodes and the total duration of immobility. The amount of time that each mouse remained immobile was recorded in seconds and then expressed as a percentage of the total time or as a percentage per minute. In this test, the ‘immobile period’ was defined as the period when the animals stopped struggling for ≥ 1 s. Data acquisition and analysis were performed using the ANY-MAZE software (Stoelting Co., Ltd.).

**Figure 3:**
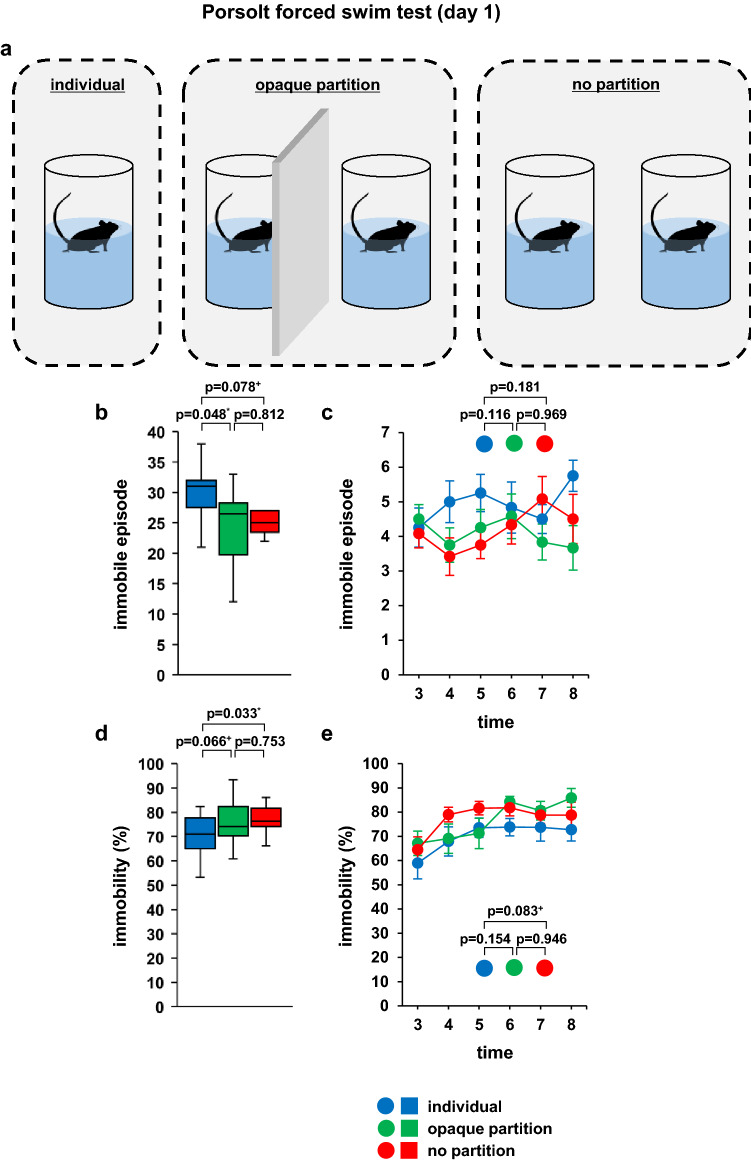
Simultaneous Porsolt forced swim test of two mice on day 1. (**a**) Schematic diagram of the experiment. Graphs showing the total number of immobility episodes (**b**) and the immobility episodes during each 1-min period (**c**). Graphs showing the total time spent immobile (**d**) and the proportion of time spent immobile during each 1-min period (**e**). All data are presented as box plots (**b**, **d**) or means ± standard errors (**c**, **e**). Statistical significance is represented by asterisks: *p < 0.05, ^+^*p* < 0.1. The *p* values were calculated using one-way analysis of variance (ANOVA) in (**b**) and (**d**) and two-way repeated-measures ANOVA in (**c**) and (**e**). SEM: standard error of the mean. individual: n = 10, opaque partition: n = 10, no partition: n = 10. Respective mean and SEM are listed in Table 2.

Three patterns were used to assess whether the behaviour of the test mice was influenced by the experimental conditions, in the same way as for the tail suspension test (Fig. 3a).

### Effect of the demonstrator in the Porsolt forced swim test

Five patterns were used to assess whether the behaviour of the test mice changed depending on the experimental conditions. We examined whether the test mice exhibited increased immobility time when allowed to observe cage mates displaying high immobility (high immobility group) or low immobility (imipramine group), and compared the results to those resulting from observing control cage mates (Fig. 5a). We also examined whether the test mice exhibited increased immobility when observing an stationary black toy (3 × 3 × 7 cm) visually similar to a mouse displaying depressive-like behaviour (stationary black toy group) or a moving black toy visually similar to an hyperactive mouse (moving black toy group) (Fig. 5a). To elicit depression-like behaviour, mice were placed in a cylinder for 10 min, and those showing depressive-like behaviour were used as demonstrators. For the imipramine group, imipramine (FUJIFILM Wako, Tokyo, Japan) was dissolved in saline to a final concentration of 5 mg/mL. Mice were injected intraperitoneally (i.p.) with 5 mg/kg imipramine 30 min before the test^22^. Vehicle control mice were injected with saline. We confirmed during the test that the high immobility group had an immobility time rate of 80% and the imipramine group had an immobility time rate lower than 60%. No dividers were installed between the demonstrators and the test subjects. Five patterns were used: (1) the cage mate was placed adjacent to the test mouse (control), (2) the cage mate with depression-like behaviour was placed adjacent to the test mouse (high immobility group), (3) the imipramine-treated hyperactive cage mate was placed adjacent to the test mouse (imipramine group), (4) the stationary black toy was placed adjacent to the test mouse (stationary black toy group), and (5) the moving black toy was placed adjacent to the test mouse (moving black toy group) (Fig. 5a).

### Statistical analyses

Statistical analyses were performed using SPSS software (IBM Corp, Tokyo, Japan). Data was analysed using one-way analysis of variance (ANOVA) followed by Tukey’s test, two-way repeated-measures ANOVA followed by Fisher’s least significant difference (LSD) test, or Student’s *t*-test. Differences were considered statistically significant at a *p* value < 0.05. All data is presented as box plots or mean ± standard error.

### Ethics approval and consent to participate

All animal experiments were performed in accordance with the U.S. National Institutes of Health (NIH) Guide for the Care and Use of Laboratory Animals (NIH Publication No. 80–23, revised in 1996) and approved by the Committee for Animal Experiments at the Kawasaki Medical School Advanced Research Centre.

## Results

### Behaviour of two mice simultaneously undergoing the tail suspension test

There were no significant differences in the number of immobility episodes or the immobile episode at 1 min between the three experimental groups (Fig. 2b; *F*_2,33_ = 0.461, *p* = 0.635, **c**; *F*_10,165_ = 1.23, *p* = 0.277, Table 1). There was no significant difference between the three groups in the percentage of time spent immobile (Fig. 2d; F_2,33_ = 0.478.0, *p* = 0.624, **e**; *F*_10,165_ = 0.659, *p* = 0.761, Table 1).

**Table 1:** Mean and SEM for Fig. 2.

			mean	SEM
Figure 2				
b	Individual		16.2	1.2
	Opaque partition		16.7	1.4
	No partition		17.8	1.1
c	Individual	3 min	4.1	0.4
		4 min	3.3	0.4
		5 min	2.7	0.3
		6 min	1.8	0.3
		7 min	1.9	0.3
		8 min	2.4	0.4
	Opaque partition	3 min	3.6	0.3
		4 min	3.3	0.6
		5 min	2.6	0.5
		6 min	2.5	0.3
		7 min	2.8	0.5
		8 min	1.9	0.3
	No partition	3 min	3.8	0.5
		4 min	3	0.2
		5 min	2.8	0.3
		6 min	2.8	0.5
		7 min	3.3	0.4
		8 min	2.2	0.3
d	Individual		80.4	2.5
	Opaque partition		83.2	2
	No partition		81.6	1.3
e	Individual	3 min	69.7	4.8
		4 min	78.9	4.5
		5 min	85.9	2.3
		6 min	78.4	8.2
		7 min	87.9	2.8
		8 min	81.7	3.4
	Opaque partition	3 min	71.8	5.4
		4 min	78.9	5.3
		5 min	87.6	2.5
		6 min	87	2.3
		7 min	85.1	2.4
		8 min	88.5	2.1
	No partition	3 min	74.6	4.6
		4 min	79	3.7
		5 min	85	1.8
		6 min	77.6	3.3
		7 min	84.2	2.3
		8 min	89.1	1.8

### Behaviour of two mice simultaneously undergoing the Porsolt forced swim test

The total number of immobility episodes was significantly lower in the opaque partition group than in the individual group (Fig. 3b; *F*_2,33_ = 2.518, *p* = 0.960, Table 2), and tended to be lower in the no-partition group than in the individual group (Fig. 3b). The total immobility time was significantly longer in the no-partition group than in the individual group (Fig. 3d; *F*_2,33_ = 2.878, *p* = 0.071, Table. 2), and tended to be longer in the opaque group than in the individual group (Fig. 3d). We found no significant differences in the immobile episodes in each 1-min period among the three groups (Fig. 3c; *F*_10,165_ = 1.208, *p* = 0.289, Table. 2), although it tended to be longer in the no-partition group than in the individual group (Fig. 3e; *F*_10,165_ = 0.726, *p* = 0.491, Table. 2).

**Table 2:** Mean and SEM for Fig. 3.

			mean	SEM
Figure 3				
b	Individual		29.6	1.6
	Opaque partition		24.6	1.8
	No partition		25.2	1.8
c	Individual	3 min	4.3	0.6
		4 min	5	0.6
		5 min	5.3	0.5
		6 min	4.8	0.7
		7 min	4.5	0.4
		8 min	5.8	0.4
	Opaque partition	3 min	4.5	0.4
		4 min	3.8	0.5
		5 min	4.3	0.5
		6 min	4.6	0.6
		7 min	3.8	0.5
		8 min	3.7	0.6
	No partition	3 min	4.1	0.4
		4 min	3.4	0.5
		5 min	3.8	0.4
		6 min	4.3	0.6
		7 min	5.1	0.6
		8 min	4.5	0.7
d	Individual		70.1	2.6
	Opaque partition		76.4	2.6
	No partition		77.4	1.7
e	Individual	3 min	58.9	6.5
		4 min	67.9	6
		5 min	73.6	4
		6 min	73.8	3.6
		7 min	73.7	5.7
		8 min	72.8	4.7
	Opaque partition	3 min	67.1	5
		4 min	69.1	6.1
		5 min	71.2	6.2
		6 min	84.2	2.3
		7 min	80.6	3.9
		8 min	85.9	3.9
	No partition	3 min	64.5	5.3
		4 min	79	3
		5 min	81.7	2.8
		6 min	81.8	3.4
		7 min	78.8	3.4
		8 min	78.7	5.4

**Figure 4:**
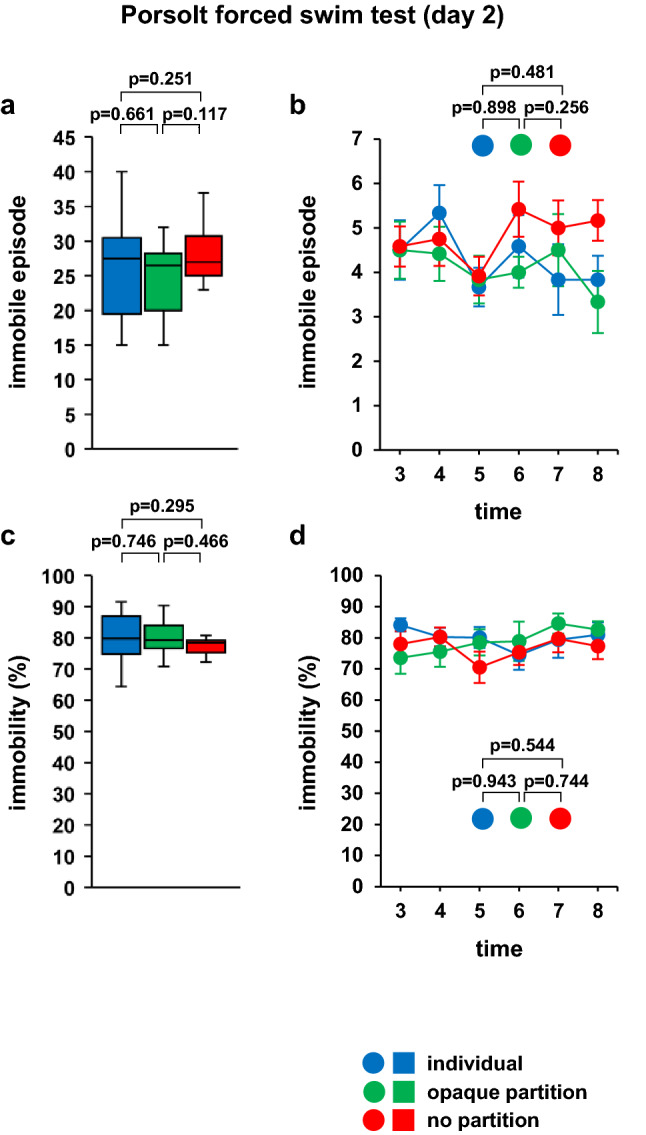
Simultaneous Porsolt forced swim test of two mice on day 2. Graphs showing the total number of immobility episodes (**a**) and the immobility episodes during each 1-min period (**b**). Graphs showing the total time spent immobile (**c**) and the proportion of time spent immobile during each 1-min period (**d**). All data are presented as box plots (**a**, **c**) or means ± standard errors (**b**, **d**). Statistical significance is represented by asterisks: *p < 0.05, ^+^*p* < 0.1. The *p* values were calculated using one-way analysis of variance (ANOVA) in (**a**) and (**c**) and two-way repeated-measures ANOVA in (**b**) and (**d**). SEM: standard error of the mean. individual: n = 10, opaque partition: n = 10, no partition: n = 10. Respective mean and SEM are listed in Table 3.

On day 2, there were no significant differences in the total immobile episodes and immobile episodes in each 1 min between the three experimental groups (Fig. 4a; *F*_2,33_ = 1.381, *p* = 0.266, b; *F*_10,165_ = 0.740, *p* = 0.686, Table. 3). There was no significant difference between the three groups in the percentage of time spent immobile (Fig. 4c; *F*_2,33_ = 0.592, *p* = 0.559, d; *F*_10,165_ = 0.827, *p* = 0.603, Table 3).

**Table 3:** Mean and SEM for Fig. 4.

			mean	SEM
Figure 4				
a	Individual		25.8	2.3
	Opaque partition		24.6	1.7
	No partition		28.8	1.6
b	Individual	3 min	4.5	0.7
		4 min	5.3	0.6
		5 min	3.7	0.4
		6 min	4.6	0.7
		7 min	3.8	0.8
		8 min	3.8	0.5
	Opaque partition	3 min	4.5	0.6
		4 min	4.4	0.6
		5 min	3.8	0.5
		6 min	4	0.3
		7 min	4.5	0.8
		8 min	3.3	0.7
	No partition	3 min	4.6	0.5
		4 min	4.8	0.6
		5 min	3.9	0.4
		6 min	5.4	0.6
		7 min	5	0.6
		8 min	5.2	0.5
c	Individual		79.9	2.5
	Opaque partition		78.9	2.2
	No partition		76.8	1.2
d	Individual	3 min	84.1	2.1
		4 min	80.2	3
		5 min	80.1	3.4
		6 min	74.5	4.8
		7 min	79.3	7.8
		8 min	80.9	4
	Opaque partition	3 min	73.5	5.1
		4 min	75.5	4.9
		5 min	78.5	4.2
		6 min	78.9	6.3
		7 min	84.6	3.2
		8 min	82.6	2.8
	No partition	3 min	78	4.2
		4 min	80.3	3.1
		5 min	70.5	5
		6 min	75.3	4
		7 min	79.6	4.2
		8 min	77.3	4.1

### Effect of the demonstrator inside the cylinder in the Porsolt forced swim test on behaviour

**Figure 5:**
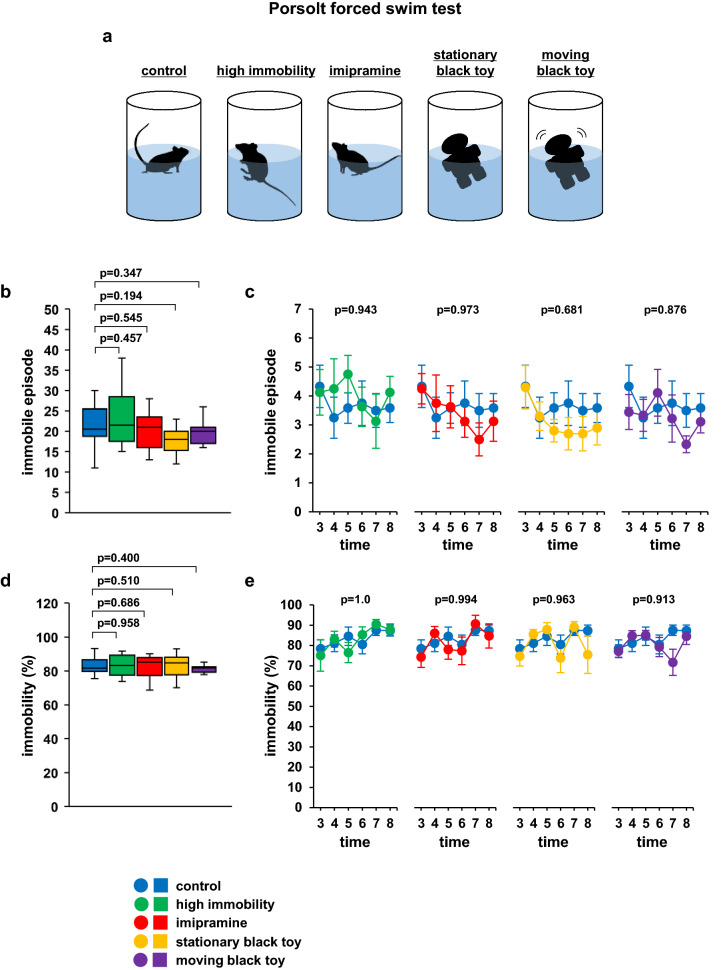
Behaviour due to the nature of the demonstrator inside the cylinder in the Porsolt forced swim test. (**a**) Schematic diagram of the experiment. Graphs showing the total number of immobility episodes (**b**) and the immobility episodes during each 1-min period (**c**). Graphs showing the total time spent immobile (**d**) and the proportion of time spent immobile during each 1-min period (**e**). All data are presented as box plots (**b**, **d**) or means ± standard errors (**c**, **e**). Statistical significance is represented by asterisks: *p < 0.05, ^+^*p* < 0.1. The *p* values were calculated using one-way analysis of variance (ANOVA) in (**b**) and (**d**) and two-way repeated-measures ANOVA in (**c**) and (**e**). SEM: standard error of the mean. control: n = 10, high immobility: n = 10, imipramine: n = 10, stationary black toy: n = 10, moving black toy: n = 10. Respective mean and SEM are listed in Table 4.

In the Porsolt forced swim test, we found no significant differences in the total number of immobility episodes (Fig. 5b; *F*_4,42_ = 1.160, *p* = 0.342, Table 4) and the immobility episode in each 1-min period among the five groups (Fig. 5c; *F*_20,210_ = 0.375, *p* = 0.991, Table 4). There were no significant differences in the total immobility time and immobility time in each 1-min period between the five experimental groups (Fig. 5d; *F*_4,42_ = 0.257, *p* = 0.904, e; *F*_20,210_ = 1.313, *p* = 0.173, Table 4).

**Table 4:** Mean and SEM for Fig. 5.

			mean	SEM
Figure 5				
b	Control		22	1.6
	High immobility		24	3.1
	Imipramine		20.4	1.8
	Stationary black toy		18.7	1.7
	Moving black toy		19.6	1.1
c	Control	3 min	4.3	0.7
		4 min	3.3	0.7
		5 min	3.6	0.5
		6 min	3.8	0.8
		7 min	3.5	0.6
		8 min	3.6	0.5
	High immobility	3 min	4.1	0.8
		4 min	4.3	1
		5 min	4.8	0.6
		6 min	3.6	0.7
		7 min	3.1	0.9
		8 min	4.1	0.5
	Imipramine	3 min	4.3	0.5
		4 min	3.8	1
		5 min	3.6	0.7
		6 min	3.1	0.5
		7 min	2.5	0.6
		8 min	3.1	0.7
	Stationary black toy	3 min	4.3	0.7
		4 min	3.3	0.5
		5 min	2.8	0.4
		6 min	2.7	0.6
		7 min	2.7	0.6
		8 min	2.9	0.6
	Moving black toy	3 min	3.4	0.6
		4 min	3.3	0.6
		5 min	4.1	0.8
		6 min	3.2	0.8
		7 min	2.3	0.3
		8 min	3.1	0.4
d	Control		83.2	1.6
	High immobility		83.1	2.6
	Imipramine		81.8	3
	Stationary black toy		81.1	3.4
	Moving black toy		80.3	1.4
e	Control	3 min	78.5	4.3
		4 min	81.1	4.1
		5 min	84.5	4.5
		6 min	80.5	4.6
		7 min	87.5	2.6
		8 min	87.3	2.7
	High immobility	3 min	75.1	7.8
		4 min	83.1	3.9
		5 min	76.5	5
		6 min	85.3	3.8
		7 min	90.5	2.4
		8 min	87.8	2.8
	Imipramine	3 min	74.3	5.2
		4 min	86	3.3
		5 min	78	4.8
		6 min	77.3	6.8
		7 min	90.6	4.3
		8 min	84.7	6
	Stationary black toy	3 min	74.7	4.8
		4 min	85.5	2.4
		5 min	87.9	3.4
		6 min	73.8	7.2
		7 min	89.1	2.6
		8 min	75.5	9.3
	Moving black toy	3 min	77.1	3.2
		4 min	84.7	2.6
		5 min	85.1	2.4
		6 min	79.2	4.8
		7 min	71.7	6.4
		8 min	84.6	4.1

## Discussion

In this study, we investigated the effect of simultaneous testing of two mice on the immobility time displayed during the forced swim and tail suspension tests. In the tail suspension test, simultaneous testing, either with or without an opaque screen between the test subjects, showed no effect on immobility time when compared with the results of testing a single mouse at a time. In the forced swim test, the immobility time of the test mice increased when they were adjacent to a cage mate. These results indicate that the test environment in the forced swim test influences immobility time.

In the tail suspension test, there was no significant difference in the number of immobility episodes or in the immobility time between the three groups. Although mice are thought to change their behaviour in response to visual information^8–10^, they failed to change their behaviour in this experiment despite the presence of nearby cage mates. Mice experience stress and anxiety when handled by their tails^23–25^. Studies of mouse behavioural phenotypes have identified handling stress as one of the most probable causes of failure in replicating phenotypes within and between experiments^26,27^. It is possible that mice could not appropriately grasp the surrounding environment during this test due to handling-related distress. Furthermore, unlike the case in the forced swim test, mice cannot freely turn around and explore the entire visual field during the tail suspension test because the tail is fixed and cannot rotate^6^, implying that they cannot grasp the surrounding environment. In this study, the results of the tail suspension test were not affected by whether one or two mice were tested simultaneously.

In the forced swim test, the proportion of immobility time was increased in the mice tested simultaneously without a partition plate, compared with that displayed by the mice that were tested alone. The proportion of immobility time tended to increase when two mice were tested simultaneously but were separated by a partition, again compared with the mice tested alone. Mice tend to avoid water^28^, and quickly become immobile when they are placed in this medium.

Behavioural experiments in mice using water include forced swim tests, water T-maze tests, and the Morris water maze (MWM) test^29^. The MWM is a widely used model for studying learning and memory behaviour. This test specifically evaluates spatial learning and memory^30,31^. In the MWM, the experimental subject relies on distal cues to find a submerged escape platform from the starting position around the swimming pool. Thus, it is assumed that the subject can grasp the surrounding environment while floating on water. In the forced swim test used in the study, it was suggested that mice grasped the surrounding environment from visual and auditory information while floating in the water and changed their behaviour accordingly. Alternatively, mice may cease to display active swimming behaviour to understand the environment in which they are placed and spend time collecting visual and auditory information. Mice are capable of visually identifying the behaviour of cage mates and simultaneously using olfactory and auditory cues as additional sensory information^32^. In this study, in the opaque group as well as in the no-partition group, they could also use olfactory and auditory information. The results of this study suggest that it is necessary to pay attention to the interpretation of the results when performing a forced swim test with two mice simultaneously, with or without a partition plate.

There were no significant differences between the groups in the results of the forced swim test performed on the second day, when it was considered that the mouse already had a good understanding of the test environment. It has been shown that immobility time increases 7 and 14 days after the initial test^33^. Further research is needed to investigate this phenomenon and its association with memory processes.

In an experimental environment without dividers, we investigated whether the nature of the demonstrator in the adjacent transparent cylinder changed the depressive-like behaviour of the test subjects, since it has been shown that mice change their behaviour by imitation of other individuals^34–39^. However, in this experiment, the behaviour of the cage mate in the adjacent cylinder, irrespective of whether they were hyperactive or immobile, did not change the behaviour of the observer. It is likely that the mice did not have the time or mental leeway to imitate the cage mate due to anxiety and fear. It is possible that changes in mouse behaviour would have occurred if the experiment duration had been extended. Further research is required to clarify these details.

Behavioural tests in mice have long been used in research worldwide^40,41^.Most behavioural traits are sensitive to genetic, environmental, and experimental factors such as genetic background, laboratory conditions, and previous testing experience^26^.The experimenter must conduct the behavioural tests appropriately; however, in many cases, it is not possible to determine from the methodological description in the research paper in what type of environment and how each researcher actually conducted the behavioural tests. Therefore, the experimental results differ across laboratories. The results we report here may be one of the causes lead to differing experimental results from different laboratories.

Studying rodent behaviour contributes to the understanding of the pathophysiology of neuropsychiatric disorders and opens new avenues for treatment. Over time, rodent behavioural tests have continued to evolve, with over 100 tests currently in use^42^. Unfortunately, laboratory behavioural testing of rodents has proven difficult, and test results may vary depending on the person conducting the experiment, the laboratory in which the experiment was conducted, and the environment^26,43^. As there is no perfect behavioural test, the best option should be chosen according to the specific project goals. To understand the behaviour of animals better, it is important to recognise that their worldview may differ significantly from that of humans^44^. As shown in this study, even in the extensively used forced swim test, mice change the number of immobility episodes and the immobility time in response to slight differences in the environment. Importantly, a high immobility rate is interpreted as an increase in depressive-like behaviour. As discussed above, the increased immobility rate in forced swim tests may reflect observation-like and adaptive-like behaviours. Care must therefore be taken when interpreting the results of behavioural tests.

## Conclusion

We found that the number of immobility episodes and the total immobility time, which are indicators of depressive-like behaviour, changed when two mice were tested at the same time during the forced swim test instead of being tested alone. The results of this study emphasise the need to consider test conditions carefully when interpreting the behavioural test results of the forced swim test.

## Data Availability

The dataset is available on reasonable request from the corresponding author.

## Abbreviations

ANOVA: Analyses of variance
MWM: Morris water maze
PS: Porsolt forced swim test
TS: Tail suspension test
